# Potential of Natural Products in the Inhibition of Adipogenesis through Regulation of PPARγ Expression and/or Its Transcriptional Activity

**DOI:** 10.3390/molecules21101278

**Published:** 2016-09-23

**Authors:** Shi Feng, Laura Reuss, Yu Wang

**Affiliations:** Citrus Research and Education Center, Food Science and Human Nutrition, University of Florida, 700 Experiment Station Road, Lake Alfred, FL 33850, USA; fsophias@ufl.edu (S.F.); lereuss@ufl.edu (L.R.)

**Keywords:** obesity, adipogenesis, PPARγ, natural products, PPARγ expression, PPARγ transcriptional activity

## Abstract

Obesity is a global health problem characterized as an increase in the mass of adipose tissue. Adipogenesis is one of the key pathways that increases the mass of adipose tissue, by which preadipocytes mature into adipocytes through cell differentiation. Peroxisome proliferator-activated receptor γ (PPARγ), the chief regulator of adipogenesis, has been acutely investigated as a molecular target for natural products in the development of anti-obesity treatments. In this review, the regulation of PPARγ expression by natural products through inhibition of CCAAT/enhancer-binding protein β (C/EBPβ) and the farnesoid X receptor (FXR), increased expression of GATA-2 and GATA-3 and activation of the Wnt/β-catenin pathway were analyzed. Furthermore, the regulation of PPARγ transcriptional activity associated with natural products through the antagonism of PPARγ and activation of Sirtuin 1 (Sirt1) and AMP-activated protein kinase (AMPK) were discussed. Lastly, regulation of mitogen-activated protein kinase (MAPK) by natural products, which might regulate both PPARγ expression and PPARγ transcriptional activity, was summarized. Understanding the role natural products play, as well as the mechanisms behind their regulation of PPARγ activity is critical for future research into their therapeutic potential for fighting obesity.

## 1. Introduction

In recent decades, the prevalence of obesity has received serious attention worldwide. This chronic disease poses a severe threat to overall human health, as it increases the risk for a myriad of clinical conditions that include diabetes, hypertension, coronary atherosclerotic heart disease and certain cancers. Obesity can develop from genetic predisposition, an individual’s metabolism, diet, lack of physical activity, as well as social status and lifestyle [1]. It is characterized by an increase in the mass of adipose tissue, specifically white adipose tissue. There are two major types of adipose tissues in mammals: white adipose tissue and brown adipose tissue. White adipose tissue plays a crucial role in lipid homeostasis and maintaining energy balance. It also stores energy in the form of triglycerides. The development of white adipose tissue is lifelong [2]. Unlike white adipose tissue, brown adipose tissue is especially abundant in newborns [3], but tends to decrease as humans age [4]. Brown adipose tissue is responsible for thermoregulation and heat production through non-shivering thermogenesis. An increase in the mass of adipose tissue can arise by increasing cell size (hypertrophy), cell number (hyperplasia or adipogenesis) or both. Adipogenesis is the process by which preadipocytes mature into adipocytes through cell differentiation. Adipocyte number does not necessarily promote obesity directly. Instead, the adipocyte number set during childhood and adolescence seems to play a major role in determining the lipid-storing capacity of adipose tissue and fat mass in adults [5]. Therefore, regulation of adipogenesis may be one critical pathway for controlling or reversing obesity. Furthermore, identifying potential adipogenic molecular targets that can be modulated by external factors, such as food and drug agents from natural products, may largely contribute to the treatment of obesity. Peroxisome proliferator-activated receptor γ (PPARγ), the chief regulator of adipogenesis, has been acutely investigated as a molecular target for natural products in the development of anti-obesity treatments. Understanding the role natural products play, as well as the mechanisms behind their inhibition of PPARγ activity is critical for future research into their therapeutic potential for fighting obesity. This review summarizes the current knowledge of anti-adipogenesis mechanisms and the corresponding natural products that exhibit inhibitory effects of PPARγ activity. Studies of adipogenesis in white adipose tissue will be the focus of this paper.

## 2. PPARγ and Its Role in Adipogenesis

The peroxisome proliferator-activated receptors (PPARs) belonging to the thyroid/retinoid nuclear receptor family are transcription factors activated by lipophilic hormones, including steroids, thyroid hormones and vitamin A metabolites [6]. Primarily, PPARs control the expression of gene networks occurring in adipogenesis, lipid metabolism, inflammation and the maintenance of metabolic homeostasis [7]. They are also involved in the pathology of various diseases, such as obesity, type 2 diabetes, dyslipidemia and inflammatory conditions [8]. Among PPARs, the peroxisome proliferator-activated receptor gamma (PPARγ), predominately expressed in adipose tissue, has been identified as a critical modulator that is not only sufficient, but also necessary for adipogenesis [9]. The importance of PPARγ for adipocyte development and function was shown both in vitro and in vivo [10,11]. It was observed that ectopic expression of PPARγ in NIH-3T3 fibroblasts caused a large percentage of these cells to undergo adipogenesis [12]. Furthermore, PPARγ added to cultured myoblasts resulted in lipid accumulation and expression of adipocyte-specific markers [13]. In addition, mice chimeric for wild-type and PPARγ null cells exhibited little or no contribution to adipose tissue by null cells [14]. It was also observed that conditional ablation of PPARγ in mature adipocytes led to cell death and subsequent repopulation by PPARγ-positive preadipocytes [15]. Moreover, humans with dominant-negative mutations introduced into the ligand-binding domain of PPARγ displayed abnormal body fat distribution [16,17].

Activation of PPARγ by certain agents has been demonstrated to stimulate adipogenesis in vitro and in vivo. It is believed that most pro-adipogenic factors function through activation of PPARγ expression or at least PPARγ activity to some extent [18]. PPARγ exists in three major protein isoforms, PPARγ1, PPARγ2 and PPARγ3 [19,20]. While PPARγ1 is broadly expressed, PPARγ2 is almost adipocyte specific [21]. PPARγ3 is expressed in adipose tissue, as well as macrophages [20]. Additionally, PPARγ2 possesses an additional stretch of 30 amino acid residues in the ligand-independent domain of the N-terminal, which leads to higher transcriptional activity as compared to PPARγ1 and PPARγ3 [19,20,22]. PPARγ is induced during adipocyte differentiation and before transcriptional activation of most adipocyte genes. It cooperates with other transcriptional factors, especially the CCAAT/enhancer binding proteins (C/EBPs) [23], to regulate adipocyte differentiation. C/EBPβ and C/EBPδ induce the expression of PPARγ and C/EBPα at the initial stage of adipogenesis, and subsequently, the concerted action of both PPARγ and C/EBPα is required during the terminal differentiation of adipocytes. Moreover, it is proposed that PPARγ is not only essential for adipogenesis, but also indispensable in the maintenance of the differentiated state [18]. Functioning as an essential regulator of adipogenesis, PPARγ has been a critical target in the design and development efforts of numerous anti-obesity drugs. PPARγ can be directly regulated by certain natural products whereas additional natural sources can also target and modulate upstream regulators of PPARγ. This review investigates the regulation of PPARγ expression by natural products through inhibition of CCAAT/enhancer-binding protein β (C/EBPβ) and farnesoid X receptor (FXR), the increased expression of GATA-2 and GATA-3 and the activation of the Wnt/β-catenin pathway. Furthermore, the regulations of PPARγ transcriptional activity associated with natural products through antagonism of PPARγ and activation of Sirtuin 1 (Sirt1) and AMP-activated protein kinase (AMPK) were discussed. Lastly, the regulation of mitogen-activated protein kinase (MAPK) by natural products, which might regulate both PPARγ expression and PPARγ transcriptional activity, was summarized.

## 3. Natural Product Regulation of PPARγ

**Figure 1:**
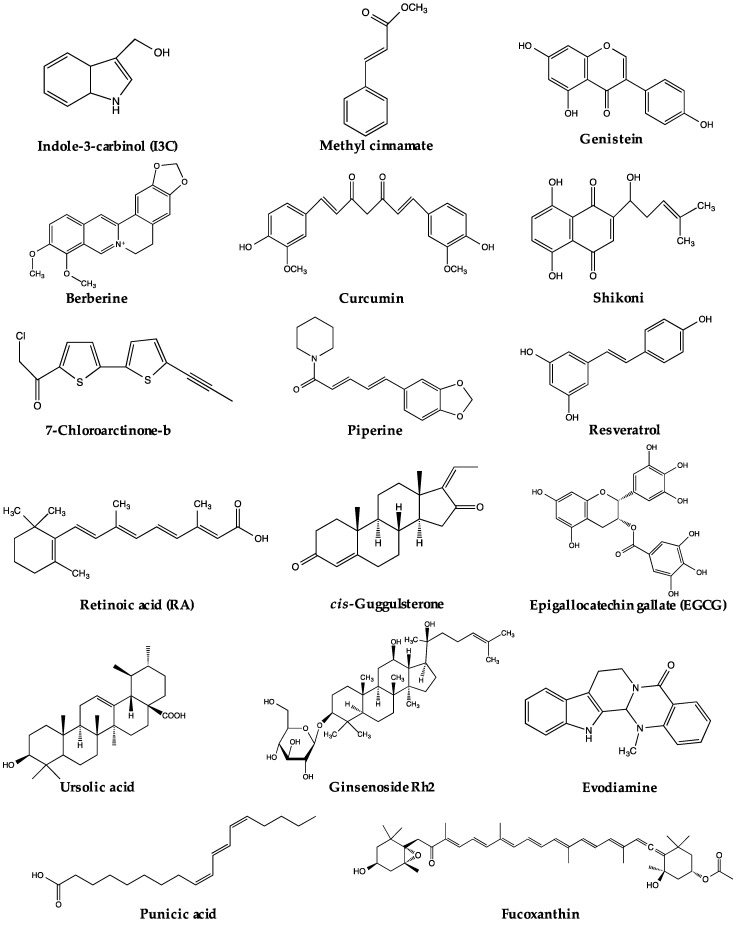
Chemical structures of active compounds regulating PPARγ derived from natural products.

**Figure 2:**
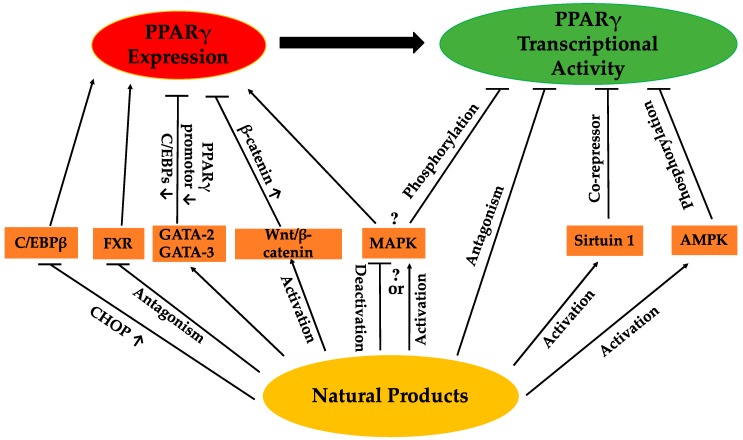
Possible pathways by which natural products regulate PPARγ.

The regulation of PPARγ can be carried out directly or/and indirectly via the anti-adipogenic activities of various natural products. The expression and activity of PPARγ make two straightforward targets for regulation. Thus far, a range of natural products has been reported to suppress adipogenesis via downregulation of PPARγ expression and/or activity (Table 1). Some of these natural products have been closely investigated, and certain active compounds (Figure 1) have been, at least, partly associated with the regulation of PPARγ upstream modulators or PPARγ itself. The possible pathways regulating PPARγ reviewed in this paper are listed in Figure 2. Nevertheless, for many natural products, the mechanisms regulating PPARγ remain unclear.

### 3.1. Regulation of PPARγ Expression

**Figure 3:**
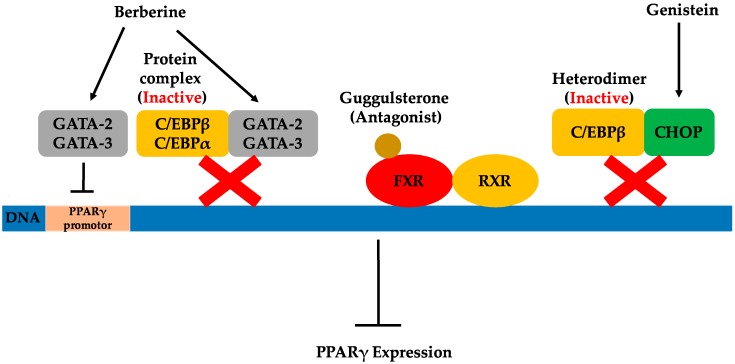
Possible pathways by which natural products regulate PPARγ expression.

The regulation of PPARγ expression by natural products through different possible pathways is demonstrated in Figure 3.

#### 3.1.1. Inhibition of CCAAT/Enhancer-Binding Protein β

C/EBPs are a family of transcription factors controlling the differentiation of a variety of cell types. C/EBPα and C/EBPβ are not only the most widely-expressed, but also the most well-studied isoforms found in the C/EBP family [65]. C/EBPα and C/EBPβ are well known for their adipogenic transcriptional activities that promote adipogenesis. Conversely, some members of the C/EBP family can repress adipocyte differentiation by forming inactive heterodimers with C/EBPα and C/EBPβ [24]. C/EBP homologous protein (CHOP) interacts with the C/EBP transcription factors to form heterodimers that cannot bind DNA, but instead function as a dominant-negative inhibitor of gene transcription [66,67]. Therefore, increasing the CHOP level may possibly inhibit C/EBPβ activity. As previously mentioned, C/EBPβ is key for inducing the initial expression of PPARγ during adipogenesis. Consequently, the direct inhibition of C/EBPβ activity can subsequently lead to PPARγ suppression, thus inhibiting adipogenesis.

Genistein, an isoflavone primarily found in legumes, was shown to have anti-adipogenic effects in 3T3-L1 cells by blocking the DNA binding and transcriptional activity of C/EBPβ [24]. This, in turn, inhibited the protein expression of differentiation-induced PPARγ and C/EBPα. The proposed mechanisms for this were a deactivation of C/EBPβ through increased levels of CHOP, as well as inhibition of the tyrosine phosphorylation of C/EBPβ. Besides anti-adipogenic effects, the pharmacological activities of genistein have been demonstrated in various published investigations and include tyrosine kinase inhibition, chemoprotective activities against cancers and cardiovascular disease and phytoestrogen activities [68]. As the simplest biosynthetic isoflavonoid compound in legumes, genistein (4′,5,7-trihydroxyisoflavone) plays the role of the central intermediary in the biosynthesis of more complex isoflavonoids. Among isoflavonoids, isoflavones including genistein are a group of compounds considered as important as the phytoestrogens that play a beneficial role in fighting obesity. Several studies of obese humans and animals indicate that phytoestrogens have significant anti-obesity effects [69,70,71]. Dietary sources of phytoestrogens include legumes, seeds and whole grains, all of which may contribute greatly to anti-obesity drug development. In addition to genistein, some other natural products have been shown to inhibit C/EBPβ activity, as well. However, the mechanism for this suppression has not been elucidated. For instance, retinoic acid (RA) was illustrated to inhibit adipogenesis by blocking C/EBPβ-induced expression of the downstream gene, PPARγ [25]. RA is considered the most active form of vitamin A, existing in the body in various essential life processes [72]. Sources rich in vitamin A include animal liver, red capsicum, sweet potato, carrots and broccoli. More recently, the extract of *Rehmannia glutinosa*, a traditional Chinese medicine, was demonstrated to inhibit adipocyte differentiation through the inhibition of C/EBPβ expression [26].

#### 3.1.2. Inhibition of the Farnesoid X Receptor

The farnesoid X receptor, important in both bile acid and cholesterol homeostasis, was first identified in 1999 as a nuclear receptor for bile acids [73]. FXR expression has been reported in liver, intestine, kidney and the adrenal glands [74]. Recently, FXR expression was reported in adipose tissue, thus the role of FXR regulation of adipogenesis in 3T3-L1 cells was investigated [75]. FXR was demonstrated to promote adipocyte differentiation by at least partially inducing PPARγ2 and C/EBPα gene expressions in in vivo experiments. It regulated adipogenesis by both PPARγ-dependent and independent mechanisms. The process of FXR activation is similar to other nuclear hormone receptors. An agonist ligand is required for FXR activation after heterodimerization of the retinoid X receptor (RXR). Then, FXR regulates target gene expression by binding to FXR response elements. Therefore, investigating antagonist ligands for FXR is one approach for fighting obesity through inhibition of FXR activity and, thereby, suppressing PPARγ2 expression.

An early study of the plant sterol guggulsterone, extracted from the resin of the guggul tree, identified a highly efficacious antagonist of FXR [27]. It was demonstrated that guggulsterone exhibited cholesterol-lowering activity based on its inhibition of FXR activation [27]. More recently, guggulsterone was used to illustrate that FXR antagonism prevented preadipocyte differentiation [75]. Later, treatment with *cis-*guggulsterone during the maturation period of adipocytes was illustrated to downregulate the adipocyte-specific transcription factors PPARγ2, C/EBPα and C/EBPβ in 3T3-L1 cells [76]. In addition to studies of the individual use of guggulsterone, the combination of guggulsterone and genistein was shown to have enhanced anti-adipogenic effects in 3T3-L1 adipocytes compared to the effects of individual compounds alone [77]. Similarly, it was also demonstrated that the combination of guggulsterone and xanthohumol more potently exerted anti-obesity effects than additive effects of the individual compounds [78]. This suggests that reasonable combinations of natural products exhibiting anti-obesity effects are a possible strategy for the development of effective obesity treatments.

#### 3.1.3. Increase Expression of GATA-2 and GATA-3

The GATA family of transcription factors binds specifically to the consensus DNA sequence (A/T)GATA(A/G) and share highly conservative zinc finger DNA binding domains [79]. They play important roles in a variety of developmental processes, including adipogenesis. Among the GATA transcription factors, GATA-2 and GATA-3 are predominantly present in white adipose tissue, specifically the preadipocytes. GATA-2 and GATA-3 are considered potential preadipocyte markers and significantly contribute to the regulation of adipocyte differentiation. It has been demonstrated that the constitutive expression of GATA-2 and GATA-3 inhibits adipocyte differentiation, trapping cells in the preadipocyte stage. This can be attributed to GATAs’ ability to decrease PPARγ2 expression through direct suppression of the PPARγ2 promoter [79], as well as by interacting with the C/EBP family of transcription factors [80]. Both GATA-2 and GATA-3 can form protein complexes with either C/EBPα or C/EBPβ, interfering with their adipocytic functions. These findings indicate that GATA-2 and GATA-3 play a crucial role as molecular gatekeepers during the onset of terminal adipocyte differentiation and, therefore, may serve as targets for the therapeutic intervention of obesity.

Berberine is an isoquinoline derivative alkaloid isolated from many traditional Chinese medicinal herbs. Purified from *Cortidis rhizome*, berberine was shown early on to inhibit PPARγ by affecting the mRNA and protein levels of PPARγ, as well as PPARγ transcriptional activity in 3T3-L1 cells [81]. It was suggested that the inhibition of PPARγ by berberine is, at least partly, dependent on the C/EBPβ signal, an upstream modulator of PPARγ. The inhibition of PPARγ transcription has been demonstrated to be the result of reducing PPARγ protein levels while eliminating the possibility of berberine as the antagonist of PPARγ. More recently, a new pathway related to the inhibitory effects of berberine on adipocyte differentiation was discussed [28]. In addition to decreasing the expression of PPARγ, berberine showed the capability to increase GATA-2 and GATA-3 mRNA and protein expression along with the inhibition of adipocyte differentiation in 3T3-L1 cells. In this study, berberine was shown to influence GATA-2 and GATA-3 expression during differentiation and induced increased gene expression of these genes in a dose-dependent manner. Similar to GATA mRNA expression, a significant increase of GATA protein expression was observed in cells grown in the presence of berberine. However, the potential mechanism by which berberine may affect the expression of GATA-2 and GATA-3 was not investigated here, and further elucidation behind this observation is needed. The inhibitory effects of berberine on adipogenesis in high-fat diet-induced obese mice were also reported by the same group of researchers. Berberine was shown to reduce weight gain and food intake in high-fat diet-induced obese mice, while no effect was observed in mice fed a normal diet [82]. Both increased mRNA expression of GATA-2 and GATA-3 was demonstrated, as was the decreased expression of PPARγ and C/EBPα mRNA in the epididymal fat of high-fat diet-induced obese mice. Meanwhile, berberine plus another botanical alkaloid, evodiamine, were investigated for their inhibitory effect and transcriptional impact individually, as well as in combination on human white preadipocyte differentiation [83]. It was shown that berberine and evodiamine increased both the mRNA expression and the protein expression of GATA-2 and GATA-3. Berberine did not affect cell viability, while evodiamine substantially decreased cell viability. However, when used together, the viability inhibition effect of evodiamine was reversed by berberine. Based on current knowledge, berberine has been shown to exhibit anti-obesity potential both in vitro and in vivo.

#### 3.1.4. Activation of the Wnt/β-Catenin Pathway

**Figure 4:**
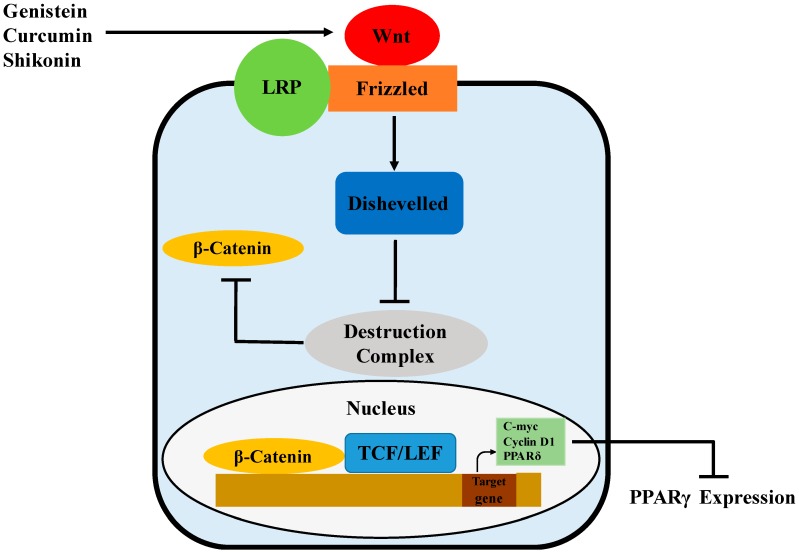
Possible mechanism of PPARγ regulation by genistein, curcumin and shikonin via activation of the Wnt/β-catenin pathway.

Wnts are a family of secreted signaling proteins that regulate cell-to-cell interactions during development and adult tissue homeostasis. Wnt signaling, likely mediated by Wnt10b, has been showed to govern adipogenesis and maintain preadipocytes in an undifferentiated state by inhibiting adipogenic transcription factors PPARγ (Figure 4) and C/EBPα [84]. This may be associated with the Wnt target genes, c-myc, cyclin D1 and PPARδ, that have been reported to inhibit the expression and activity of both PPARγ and C/EBPα [85,86,87]. Wnt signaling is initiated by the binding of Wnts to Frizzled (Fz) receptors, followed by heterodimerization between the Fz receptors and low-density lipoprotein receptor-related protein (LRP) co-receptors [88]. In the β-catenin-dependent pathway, Wnt signaling activates Disheveled, inhibits the activity of the destruction complex and thereby stabilizes β-catenin through phosphorylation [89]. The destruction complex, containing GSK-3, Axin, APC and β-TrCP/Slimb, promotes rapid degradation of β-catenin in the absence of Wnt signals. Once translocated to the nucleus, β-catenin binds to the T-cell factor/lymphoid-enhancing factor (TCF/LEF) family of transcription factors and then regulates the expression of Wnt target genes [89,90].

Genistein has been demonstrated to regulate adipogenesis through different pathways. In a recent study, repression of the adipogenic differentiation of human adipose tissue-derived mesenchymal stem cells by genistein was associated with Wnt/β-catenin signaling [29]. In this study, genistein was shown to promote Wnt signaling by interacting with Wnt ligands, Wnt antagonists and Wnt intermediates. The expression of Wnt3, which inhibits adipogenesis via regulation of PPARγ and C/EBPα expression in 3T3-L1 cells [88], was increased by genistein. Meanwhile, Wnt signaling antagonists, including sFRP1, DKK2, CK1 and Axin2, were inhibited by genistein. Genistein also augmented mRNA and protein levels of β-catenin. Curcumin, a polyphenol found in the rhizomes of *Curcuma longa*, was also observed to have anti-adipogenic effects associated with the activation of Wnt/β-catenin signaling in 3T3-L1 cells [30]. During differentiation, curcumin restored nuclear translocation of β-catenin and reduced differentiation-stimulated expression of the components in the destruction complex, including CK1α, GSK-3β and Axin. Meanwhile, curcumin was observed to increase mRNA levels of c-Myc and cyclin D1, the Wnt target genes. More recently, shikonin isolated from *Lithospermun erythrorhizon* Sieb. Et Zucc was shown to inhibit adipogenesis through modulation of the Wnt/β-catenin pathway [31]. In this study, shikonin maintained the nuclear level of β-catenin while increasing the level of its transcriptional product, cyclin D1 during adipogenesis of 3T3-L1 cells. Meanwhile, shikonin-induced reductions of PPARγ and C/EBPα were significantly recovered by siRNA-mediated knockdown of β-catenin. Similarly, isoquercitrin and isorhamnetin, active flavonoids in the extract of *Persicaria hydropiper* (L.) spach (an herbal plant used to add spicy flavor to traditional Chinese dishes), were demonstrated to inhibit the adipocyte differentiation of 3T3-L1 cells via the activation of Wnt/β-catenin signaling [91].

### 3.2. Regulation of PPARγ Transcriptional Activity

#### 3.2.1. Antagonist of PPARγ

PPARγ functions as a heterodimer with the retinoid X receptor (RXR) and binds to specific DNA sequences to regulate the transcription of target genes [7]. The activity of PPARγ is regulated by binding agonist ligands, including steroid and thyroid hormones, vitamins, lipid metabolites and xenobiotics [92]. Unliganded (agonist ligand) PPARγ/RXR heterodimers repress the transcription of target genes by interacting with corepressor molecules [93], while PPARγ binding with the agonist ligand experiences conformational changes facilitating the dissociation of corepressor molecules to enable recruitment of coactivators to the liganded receptor. Therefore, investigation of PPARγ antagonists that may directly inhibit PPARγ activity by interrupting its functional pathway can be considered a logical approach for research in the fight against obesity.

In recent years, researchers have been attempting to screen natural products for novel PPARγ antagonists with inhibitory effects on adipogenesis. 7-chloroarctinone-b (CAB), a new thiophene-acetylene type of derivative isolated from *Rhaponticum uniflorum*’s roots, was identified as a specific PPARγ antagonist [32]. The inhibitory effects of CAB on PPARγ/RXRα heterodimerization and PPARγ co-activation recruitment were studied. CAB showed high binding affinity for the PPARγ-ligand binding domain (LBD), thereby considerably antagonizing the PPARγ agonist rosiglitazone-stimulated PPAR γ-LBD/RXR α-LBD dimerization. Meanwhile, it also considerably antagonized rosiglitazone-stimulated PPARγ coactivator recruitment. In the 3T3-L1 cell differentiation assay, it was demonstrated that CAB effectively antagonized both hormone and rosiglitazone-induced adipocyte differentiation in cell culture. Thiophene-acetylene represents a unique class of natural products presenting a wide variety of biological activities, including antitumor, antiviral, anti-HIV and antifungal to insecticidal activities [94]. The discovery of PPARγ’s antagonistic activity of CAB may imply the potential of thiophene-acetylene as an anti-obesity treatment. Similarly, piperine, a major alkaloid-amine component of black pepper, was shown to inhibit adipogenesis by antagonizing PPARγ activity in 3T3-L1 cells [33]. Piperine significantly repressed the rosiglitazone-induced PPARγ transcriptional activity while also markedly decreasing mRNA expression of PPARγ. Furthermore, piperine was demonstrated to disrupt the rosiglitazone-dependent interaction between PPARγ and coactivator CREB-binding protein (CBP). Before being discovered as a potential antagonist of PPARγ, piperine had previously been known for its painkilling, antioxidant, antitumor and anti-inflammatory activities [33].

#### 3.2.2. Activation of Sirtuin 1

Sirt1 is an NAD^+^-dependent nuclear deacetylase in mammals. It has been identified as a key mediator of pathways located downstream of calorie restriction and considered a therapeutic target in the treatment of age-related diseases [95]. Sirt1 is involved in processes that include chromatin remodeling, transcriptional silencing, chromosomal stability, cell cycle progression, apoptosis, autophagy, metabolism, growth suppression, inflammation and stress responses [96]. Recently, Sirt1 has emerged as a novel therapeutic target in the fight against obesity. It has been demonstrated that Sirt1 is a negative modulator of adipogenesis in the 3T3-L1 model [97]. In 3T3-L1 adipocytes, overexpression of Sirt1 was shown to attenuate adipogenesis, while the RNA interference of Sirt1 enhanced it. Meanwhile, upregulation of Sirt1 led to lipolysis and the loss of fat in differentiated adipocytes. It is believed that Sirt1 promotes fat mobilization in white adipocytes through PPARγ repression, interacting with its cofactors nuclear receptor co-repressor (NCoR) and the silencing mediator of retinoid and thyroid hormone receptors (SMRT). Sirt1 and PPARγ have been shown to bind to the same DNA sequences, suggesting that Sirt1 plays a role as a co-repressor of PPARγ. This is consistent with a previous study where, in the absence of ligand, PPARγ recruited NCoR and SMRT, and these co-repressors were capable of downregulating PPARγ-mediated transcriptional activity [98]. 3T3-L1 cells deficient in NCoR or SMRT were exposed to differentiation media; they then exhibited increased expression of adipocyte-specific genes and increased production of lipid droplets, as compared to the control cells. Therefore, activation of Sirt1 during adipogenesis could be beneficial in the therapeutic intervention of obesity. Besides regulation of PPARγ, activated Sirt1 further deacetylates the transcriptional coactivator peroxisome proliferator-activated receptor gamma coactivator 1-alpha (PGC-1) at promoter regions to induce the expression of genes involved in mitochondrial biogenesis and fatty acid oxidation [99].

Resveratrol is a member of the natural polyphenols found in plants. It is abundant in Japanese knotweed and found in considerable amounts in peanuts, groundnuts, grapevines and red vines [100]. Resveratrol has gained much attention due to its numerous, beneficial cardio-protective effects [101] and anti-cancer [102], anti-inflammatory and antioxidant actions [103]. Recently, resveratrol has been shown to play a role in multiple activities in adipose tissue, including adipocyte proliferation, adipogenesis, lipolysis and apoptosis [104]. It is the first polyphenolic compound that has been shown to activate Sirt1 [105,106]. Moreover, resveratrol was identified as the most potent polyphenolic compound activator of Sirt1 [34,95]. It has been shown to inhibit proliferation and adipogenic differentiation in a Sirt1-dependent manner in human preadipocytes [107]. Human preadipocytes were used to demonstrate the inhibitory effect of resveratrol on adipogenic differentiation. The fact that the inhibitory activity of resveratrol was abolished by the knockdown of Sirt1 indicated the role of Sirt1 as a mediator of the resveratrol effect. Similarly, in an in vitro study, investigation of the inhibitory effects of resveratrol on adipogenesis was conducted in pig primary preadipocytes [108]. The downregulation of adipogenesis was also associated with an increased expression of Sirt1 mRNA, which, in turn, suppressed PPARγ. Thus far, the mechanisms that link resveratrol to the activation of Sirt1 are still debated, and recent studies have shown that resveratrol does not directly activate Sirt1 [109,110].

Along with resveratrol, some other polyphenols, such as butein, piceatannol and quercetin, were shown to enhance Sirt1 activity in vitro through the demonstration of the effects on the stimulation of the Sirt1 catalytic rate [34]. There has been a very limited number of studies assessing the ability of other polyphenols, including quercetin and catechins, to induce Sirt1 activity [111,112]. Some conclusions are contradictory, as both the inhibitory effects and stimulation effects have been observed, which may be explained as the powerful effects of polyphenol stability and metabolism on the stimulation of Sirt1. Xanthigen, a source of punicic acid and fucoxanthin derived from pomegranate seed and brown seaweed, was demonstrated to deregulate Sirt1 and activate AMP-activated protein kinase (AMPK) signaling accompanied with downregulation of PPARγ [36]. More recently, It was reported that indole-3-carbinol (I3C), an efficacious, specific Sirt1 activator in cultured 3T3-L1 cell lines, ameliorated adipogenesis [35]. I3C is the product of the breakdown of glucosinolate glucobrassicin, which is abundant in many vegetables, including broccoli, cabbage, cauliflower and kale. I3C did not inhibit adipocyte differentiation in 3T3-L1 cells where Sirt1 was knocked down. Furthermore, reverse transcriptase polymerase chain reaction analysis showed that I3C treatment reduced mRNA levels of adipogenic genes encoding for C/EBPα, PPARγ2, FAS and aP2 in 3T3-L1 cells, but not in Sirt1 knockdown cells. Understanding the role and mechanisms of natural products in Sirt1 regulation is crucial for the discovery and development of pharmacological agents for potential use in the clinical management of obesity.

#### 3.2.3. Activation of AMP-Activated Protein Kinase

AMPK is a serine/threonine protein kinase that works as an essential regulator of cellular metabolism and energy balance. It is a heterotrimeric protein consisting of three subunits, α, β and γ, among which the catalytic subunit α is vital for AMPK activation through its own activation via phosphorylation [113]. AMPK promotes mechanisms that increase energy production and shut down pathways that consume energy. The anti-adipogenic effects of AMPK are expected since adipocyte differentiation can be seen as an energy-consuming process involving new membrane and protein synthesis, two process that are heavily prohibited by AMPK activation [114,115]. Long-term activation of AMPK can affect the pattern of gene expression in a variety of circumstances. To be more specific, AMPK can directly phosphorylate and regulate proteins involved in gene transcription, such as transcription factors, cofactors and components of the transcriptional core machinery [116]. Activation of AMPK has been shown to attenuate the expression of PPARγ in 3T3-L1 adipocytes [117]. AMPK was observed as an upstream positive regulator of p38 MAPK [118], which was demonstrated to promote PPARγ phosphorylation, inhibit its transcriptional activity and thereby block adipocyte differentiation [119,120]. The modulation of AMPK has been viewed as a potential obesity therapy. Berberine was demonstrated to increase the activation (phosphorylation) of AMPK and P38, as well as the deactivation of PPARγ in adipocytes [121], and they were proposed as the possible pathways by which berberine mediated its metabolic actions. Curcumin was confirmed to regulate adipocyte differentiation by activating AMPK and downregulating PPARγ transcription [37]. It was observed that AMPK activation led to the inhibition of PPARγ expression in 3T3-L1 adipocytes, although the second regulator between AMPK and PPARγ was not elucidated. More recently, the anti-obesity effects of *Lysimachia foenum-graecum* (LFE) were associated with its ability to block adipogenesis in 3T3-L1 adipocytes through the possible link between LFE-induced activation of AMPK and AMPK-mediated suppression of PPARγ transcriptional activity [38].

Accumulating evidence has shown the important effects of AMPK activation by natural products in the inhibition of adipogenesis. Genistein exerted the inhibition of adipocyte differentiation through the activation of AMPK paralleled with the generation of reactive oxygen species (ROS), an upstream signal for AMPK that rapidly activates AMPK [39]. Anti-obesity effects of ginsenoside Rh2, an active component derived from *Panax ginseng*, are associated with the activation of the AMPK signaling pathway in 3T3-L1 adipocytes, while ROS plays a role in AMPK activation during ginsenoside Rh2 treatment [40]. It was also demonstrated that ursolic acid (UA), a triterpenoid compound identified in many fruits and plants, increased phosphorylation and activation of AMPK by stimulating LKB1 (an upstream kinase of AMPK) activity and consequently inhibited adipogenesis [42].

### 3.3. Comprehensive Regulation of PPARγ

#### Regulation of Mitogen-Activated Protein Kinase

Mitogen-activated protein kinases (MAPKs) are well known for their three subfamilies, ERK, JNK/SAPK and p38. The activation of MAPK occurs through phosphorylation of specific threonine and tyrosine residues on the MAPK molecule by an upstream kinase, MAPK kinase [122]. MAPK cascades are interactive intracellular signal transduction pathways that often regulate cellular control switches [123,124]. These cascades consist of a three-kinase module, MAPK, MEK (MAPK/ERK kinase, MAPK activator) and MEKK (MEK kinase, MEK activator) [122]. In an early study, activation of MAPK was shown to antagonize 3T3-L1 adipocytic differentiation [125]. It is believed that MAPK-mediated phosphorylation of PPARγ contributes to the reduction of PPARγ transcriptional activity and, thereby, inhibits adipocyte differentiation [119]. PPARγ activity is finely modulated; in addition to ligand-mediated activation, its phosphorylation status and the molecular consequences include changes in ligand affinity, DNA binding, recruitment of transcriptional cofactors, proteasome degradation, etc. [120]. By using preadipocyte cultures, evodiamine, a major alkaloidal compound in the fruit of *Evodia* *fructus*, was found to increase activation of ERK/MAPK, to reduce the expression of PPARγ and, thereby, significantly inhibit adipocyte differentiation [43].

Contradictory reports also exist. It was proposed that deactivation of ERK was necessary for inhibition of adipocyte differentiation [126,127]. Green tea polyphenol EGCG was shown to inhibit adipogenesis in 3T3-L1 adipocytes [44]. It was also proposed that the possible mechanisms behind the anti-adipogenic effects of EGCG involve mitogen-activated protein (MAP) kinase, especially the extracellular signal-regulated kinases (ERKs) that are stimulated by growth-related signals. EGCG has been reported to reduce phosphor-ERK1 and phosphor-ERK2 levels in 3T3-L1 preadipocytes [128]. Additionally, ERK1/2 phosphorylation was demonstrated to promote C/EBPα and PPARγ expression in 3T3-L1 cells [127]. Here, inhibition of MAPK seems to repress adipogenesis. A similar mechanism was also observed in other natural products. The water extract of *Hibiscus sabdariffa* L., a medicinal herb, was investigated for its inhibition of adipocyte differentiation in 3T3-L1 preadipocytes [45]. Hibiscus extract blocked the MAPK pathway by inhibiting the phosphorylation of ERK and, consequently, the expression of C/EBPα and PPARγ. Further investigation into the influences of the MAPK pathway of adipocyte differentiation is definitely needed, and clear elucidation of this mechanism could provide a potential foundation for future developments in anti-obesity drugs.

## 4. Possible Future Trends and Applications of Natural Products on Anti-Obesity

Obesity is recognized as a worldwide health crisis. It is not just a severe health concern, but also a risk factor for many chronic diseases, including diabetes, hypertension, cardiovascular diseases, as well as certain cancers. The only obesity-treatment drug currently available, Orlistat, has serious side effects that include increased blood pressure, dry mouth, constipation, headache and insomnia [129]. Sibutramine, a previous prescription drug for weight loss, was withdrawn by the U.S. Food and Drug Administration in 2010 due to an increased risk of cardiovascular adverse events. Therefore, in recent years, natural alternatives that exhibit anti-obesity potential have been widely investigated. As discussed above, natural products, which have shown large potential to regulate PPARγ expression and/or transcriptional activity, are potential therapies to suppress adipogenesis and, thereby, fight against obesity.

Currently, the majority of natural products investigated are phytochemicals obtained from dietary and medicinal plants, both of which are relatively safe sources and easy to access. Primarily obtained from vegetables and fruits, dietary phytochemicals could be employed as anti-obesity agents due to evidence indicating that they may reduce adipose tissue mass through inhibiting adipogenesis, stimulating lipolysis and inducing apoptosis of existing adipocytes. Among dietary phytochemicals, polyphenols, terpenoids, phytosterols, as well as alkaloids are common groups with active components showing anti-obesity effects [130,131]. The application of medicinal plants in treating metabolic diseases has a long history, especially in Asia. Plants have always been a source of medication, although initially, their active molecules may not be clearly identified. With an increasing understanding of obesity and its key regulator, PPARγ, investigations into the functional mechanism of PPARγ along with the effective regulators of PPARγ have been largely conducted. The identification of both the possible targets that regulate PPARγ, as well as the active components that modulate PPARγ are two key steps towards fighting obesity via the inhibition of adipogenesis, and the continuation of these elucidation efforts is imperative.

The current understanding indicates that specific natural products can target more than one known pathway that regulates PPARγ expression and/or transcriptional activity. For example, genistein has been shown to suppress PPARγ expression by both the inhibition of C/EBPβ, as well as the activation of the Wnt/β-catenin pathway, whereas it also controls PPARγ transcriptional activity through activation of AMPK during adipogenesis. This suggests that a natural product such as genistein could be a highly effective anti-obesity agent. However, further investigations into the safety, metabolism and human use of genistein as a medication are necessary. Combined treatment is an additional option for obesity therapy. For instance, the combined effect of both berberine and evodiamine on human white preadipocyte differentiation has been studied with no observable significant additive or synergetic effects of adipogenesis inhibition. Nevertheless, there is no doubt that the appropriate combination of effective natural products would help the design of novel strategies in preventing the observed current epidemic levels of obesity.

It is not surprising that all known pathways for PPARγ regulation are not limited to this review. However, the study of these numerous pathways remains in the initial stages, and the application of natural products on these pathways has yet to be investigated. For example, the deficiency of cellular retinol-binding protein type 1 (CRBP1), expressed in preadipocytes prior to the expression of PPARγ, is tightly associated with enhanced PPARγ expression and activity [132]. Overexpression of Sirt2 inhibits differentiation, accompanied by decreased expression of PPARγ [133]. It is proposed that reducing the level of Sirt2 results in an increased level of forkhead box protein O1 (FOXO1) acetylation/phosphorylation, thereby reducing the ability of FOXO1 to interact with the PPARγ promoter and repress PPARγ transcription. The high-affinity and PPARγ-specific antagonist, cyclic phosphatidic acid (cPA), stabilizes the PPARγ-SMRT corepressor complex and inhibits PPARγ-mediated gene transcription [134]. It has also been observed that the signal transducer and activator of transcription 5(STAT5) stimulates the expression of PPARγ [135]. While the understanding and discovery of new pathways for PPARγ regulation are proceeding, additional therapeutic targets for obesity treatment are presented, and the further application of possible active natural products could be conducted.

## Figures and Tables

**Table 1:** Natural products that regulate PPARγ.

Mechanism of Action	Source (Possible Natural Sources)	Active Component
Inhibition of C/EBPβ	Commercial standard (legumes) [24]	Genistein
	Not specified (animal liver) [25]	Retinoic acid (RA)
	*Rehmannia glutinosa* [26]	Not specified (alcohol extract)
Inhibition of FXR	Resin of the guggul tree [27]	Guggulsterone
Increased expression of GATA-2 and GATA-3	*Cortidis rhizome* [28]	Berberine
Activation of Wnt/β-catenin pathway	Commercial standard (legumes) [29]	Genistein
	Not specified (rhizomes of *Curcuma longa*) [30]	Curcumin
	Commercial standard (*Lithospermun erythrorhizon* Sieb. Et Zucc) [31]	Shikonin
Antagonism of PPARγ	*Rhaponticum uniflorum* [32]	7-Chloroarctinone-b
	Commercial standard (black pepper) [33]	Piperine
Activation of Sirtuin 1	Not specified (Japanese knotweed, peanut) [34]	Resveratrol
	Not specified (broccoli, cabbage) [35]	Indole-3-carbinol (I3C)
	Commercial standard (pomegranate seed oil and brown seaweed extract) [36]	Xanthigen
Activation of AMPK	Commercial standard (*Curcuma longa*) [37]	Curcumin
	*Lysimachia foenum-graecum* [38]	Not specified (ethanol extract)
	Not specified (soybean) [39]	Genistein
	Commercial standard (*Panax ginseng*) [40]	Ginsenoside Rh2
	Commercial standard (*Zanthoxylum armatum*) [41]	Methyl cinnamate
	Commercial standard (*Mirabilis jalapa*, apple) [42]	Ursolic acid
	Commercial standard (pomegranate seed oil and brown seaweed extract) [36]	Xanthigen
Regulation of MAPK	Commercial standard (*Evodia fructus*) [43]	Evodiamine
	Green tea [44]	EGCG
	*Hibiscus sabdariffa* L. [45]	Not specified (water extract)
Unknown	*Lagerstroemia speciosa* L. [46]	Not specified (hot water extract)
	*Hibiscus sabdariffa* [45]	Not specified (dried flower extract)
	*Undaria pinnatifida* [47]	Fucoxanthin and fucoxanthinol
	Commercial standard (beer hops) [48]	Xanthohumol and isoxanthohumol
	Commercial standard (red pepper) [49]	Capsaicin
	*Eriobotrya japonica* leaves [50]	Corosolic Acid
	Commercial standard (vinegar, buckwheat) [51]	o-Courmaric acid and rutin
	Commercial standard (grape and onion) [52]	Resveratrol and quercetin
	Brown algae [53]	Fucoidan
	*Monascus* [54]	Monascin and ankaflavin
	Commercial standard (onion) [55]	Quercetin
	Dry *Undaria pinnatifida* powder [47]	Amarouciaxanthin A
	*Lithospermum erythrorhizon* [56]	Shikonin
	*Ginseng* [57]	Ginsenoside Rh1
	*Caesalpinia sappan* L. [58]	Brazilein
	*C. maculata* and *C. subternata* [59]	Not specified (hot water extract)
	*Adenophora triphylla* var. japonica extract [60]	Lupenone
	*Allomyrina dichotoma larvae* [61]	Not specified (ethanol extract)
	Chickpea [62]	Isoflavones
	Not specified (*Garcinia indica*, almonds) [63]	Garcinol, pterostilbene
	Rhizome of *Coptis chinensis* [64]	Berberine, epiberberine, coptisine, palmatine, and magnoflorine
